# 

**Published:** 1862-12

**Authors:** 


### DENTAL REGISTER

### OF THE WEST.

EDITED BY

J. TAFT AND GEO. WATT.

DECEMBER,–1862

**MONTHLY:**

**Three Dollars per Annum, in advance.**

CINCINNATI:

J. TAFT, PUBLISHER.

J. Taft, Dentist, No. 56 West Fourth Street.

